# Experimental data on mechanical behavior and numerical data on tensile stress distribution of a hyperelastic Polydimethysiloxane (PDMS) based membrane for cell culture

**DOI:** 10.1016/j.dib.2020.105476

**Published:** 2020-04-07

**Authors:** Nuttapol Limjeerajarus, Mahunnop Fakkao, Sorapon Na Lampang, Thanaphum Osathanon, Prasit Pavasant, Chalida Nakalekha Limjeerajarus

**Affiliations:** a Research Center for Advanced Energy Technology, Faculty of Engineering, Thai–Nichi Institute of Technology, Bangkok 10250, Thailand; b Department of Anatomy, Faculty of Dentistry, Chulalongkorn University, Bangkok 10330, Thailand; c Department of Physiology, Faculty of Dentistry, Chulalongkorn University, Bangkok 10330, Thailand; d Excellence Center in Regenerative Dentistry, Faculty of Dentistry, Chulalongkorn University, Bangkok, Thailand; e Genomics and Precision Dentistry Research Unit, Faculty of Dentistry, Chulalongkorn University, Bangkok, Thailand

**Keywords:** Tensile stress, Polydimethysiloxane, Material constants, Cell culture, Finite element analysis

## Abstract

The data contained within this article relate to experimental data on mechanical behavior of an in-house cast hyperelastic Polydimethysiloxane (PDMS) based membrane (Silastic^Ⓡ^ T-4, Dow Corning) for cell culture, which was used as a tool for applying tensile stress on cells and tissues. With the experimentally obtained material constants, tensile stress distribution over the membrane surface was numerically assessed using a finite element analysis (FEA). The membrane area having a uniform tensile stress distribution under different strain loading conditions of 1–20% was suggested for cell culture.

Specifications table

**Table:** 

Subject	Mechanical Engineering
Specific subject area	Tensile stress, Cell culture
Type of data	Table Chart Figure
How data were acquired	Universal testing machine (UTM) (Instron 3360 series) for mechanical behavior test and ANSYS WORKBENCH R16.2 for numerical simulation
Data format	Raw Analyzed
Parameters for data collection	After casting, the hyperelastic Polydimethysiloxane (PDMS) based membrane was treated with plasma and UV radiations, and coated with gelatin
Description of data collection	The data on mechanical behavior of the membrane was experimentally measured by a UTM and presented as stress-strain curves, which can be expressed by a 2nd order polynomial mathematical model. This model was then used in FEA based numerical simulations in ANSYS WORKBENCH R16.2 in order to analyze tensile stress distribution over the membrane surface under different strain loading conditions. Model validation was conducted by comparing the tension force required to generate a 20% strain loading as measured with the UTM with that estimated by the simulation. The membrane area having uniform tensile stress distribution was suggested to be used for cell culture.
Data source location	Institution: Chulalongkorn University City/Town/Region: Bangkok Country: Thailand
Data accessibility	With the article, and Repository: Mendeley Data https://data.mendeley.com/datasets/twn9d5w2yb/draft?a=4ed7eaf9-99b5-4d1b-974b-ec28b213968a
Related research article	Y. Tantilertanant, J. Niyompanich, V. Everts, P. Supaphol, P. Pavasant, N. Sanchavanakit, Cyclic tensile force stimulates BMP9 synthesis and in vitro mineralization by human periodontal ligament cells. J. Cell Physiol. 234 (2019) 4528–4539. https://doi.org/10.1002/jcp.27257

## Value of the data

• The data provide the mechanical behavior in terms of stress-strain curves of a hyperelastic PDMS based membrane used for tensile stress loading on cultured cells.
• The material constants of a 2nd order polynomial mathematical model used to explain the stress-strain curves are given with a standard error of estimate of 0.0052.
• From the numerical data, the area of the membrane having a uniform tensile stress distribution is suggested for cell culture under different strain loading conditions of 1, 3, 5, 7, 10 and 20%.
• Researchers may use the provided material constants in an FEA simulation so as to identify the area of uniformly distributed tensile stress of such PDMS membrane for cell culture experiments under their own desired strain loading conditions.

## 1 Data description

**Fig. 1:**
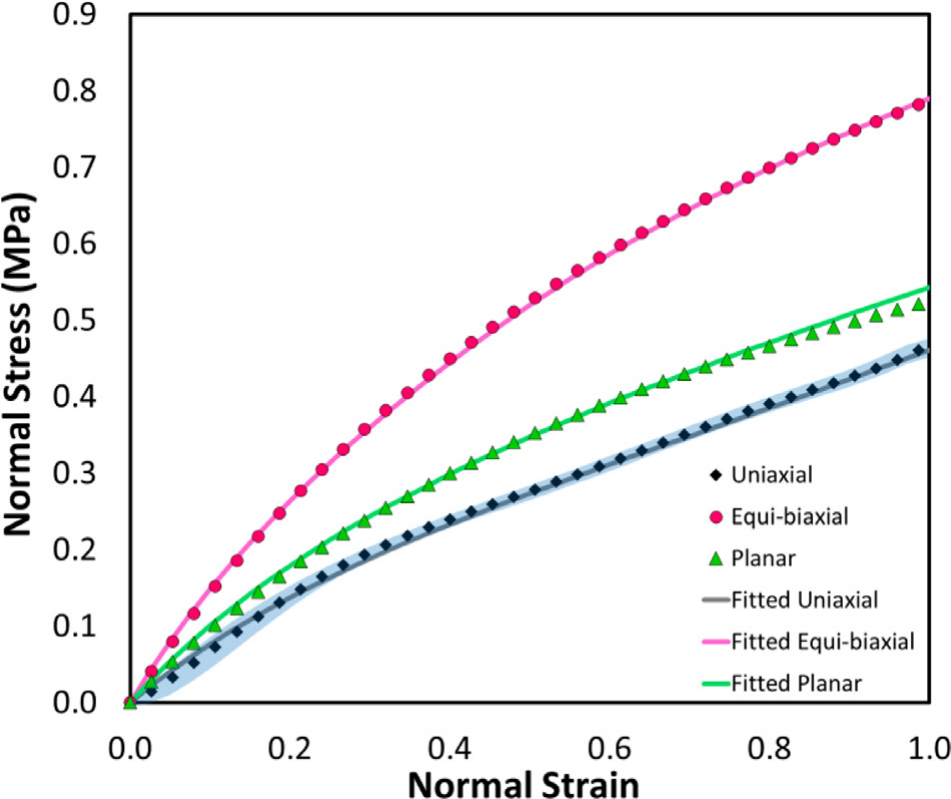
Stress-strain curves of the membrane obtained from the experiment and the 2nd order polynomial mathematical model. The shaded error band presents the standard deviation of the data.

The data presented here contain experimental data of mechanical behavior in terms of stress-strain curves and numerical data of tensile stress distribution of a hyperelastic PDMS based membrane. This membrane is used for cell culture to assess the effects of tensile stress loading on cells in vitro [1]. In Fig. 1, the average stress-strain curve for the uniaxial tensile test was experimentally obtained using a UTM. Those for equi-biaxial and planar tensile tests were estimated based on the average uniaxial data using the Turner and Brennan's model [2]. The data of these three stress-strain curves were used to identify material constants of the membrane as a function of the strain energy density (W) (Eq. (4)), which is presented in the Table 1. The dashed lines in Fig. 1 represent stress-strain curves reproduced from the fitted material constants, which had a standard error of estimate of 0.0052, as compared with the data obtained from the tensile test.

**Table 1:** The membrane material constants in the 2nd order polynomial mathematical model used in FEA.

C10	C01	C20	C11	C02
0.088525	0.047481	0.0093126	−0.006518	0.00041182

**Fig. 2:**
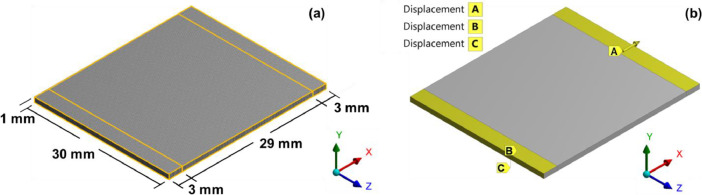
(a) The model geometry used for simulation and (b) the boundary conditions used in this model.

Fig. 2(a) presents the geometry of the membrane model used in the FEA simulation. All dimensions were the actual dimensions when the membrane was used with the linear tension loading apparatus [1]. The material constants in Table 1 were introduced in the FEA as the membrane mechanical properties. Fig. 2(b) shows boundary conditions based on the practical operating conditions under which one side was fixed and the other side (displacement A) was stretched at different strain loading conditions from 1% to 20%.

**Fig. 3:**
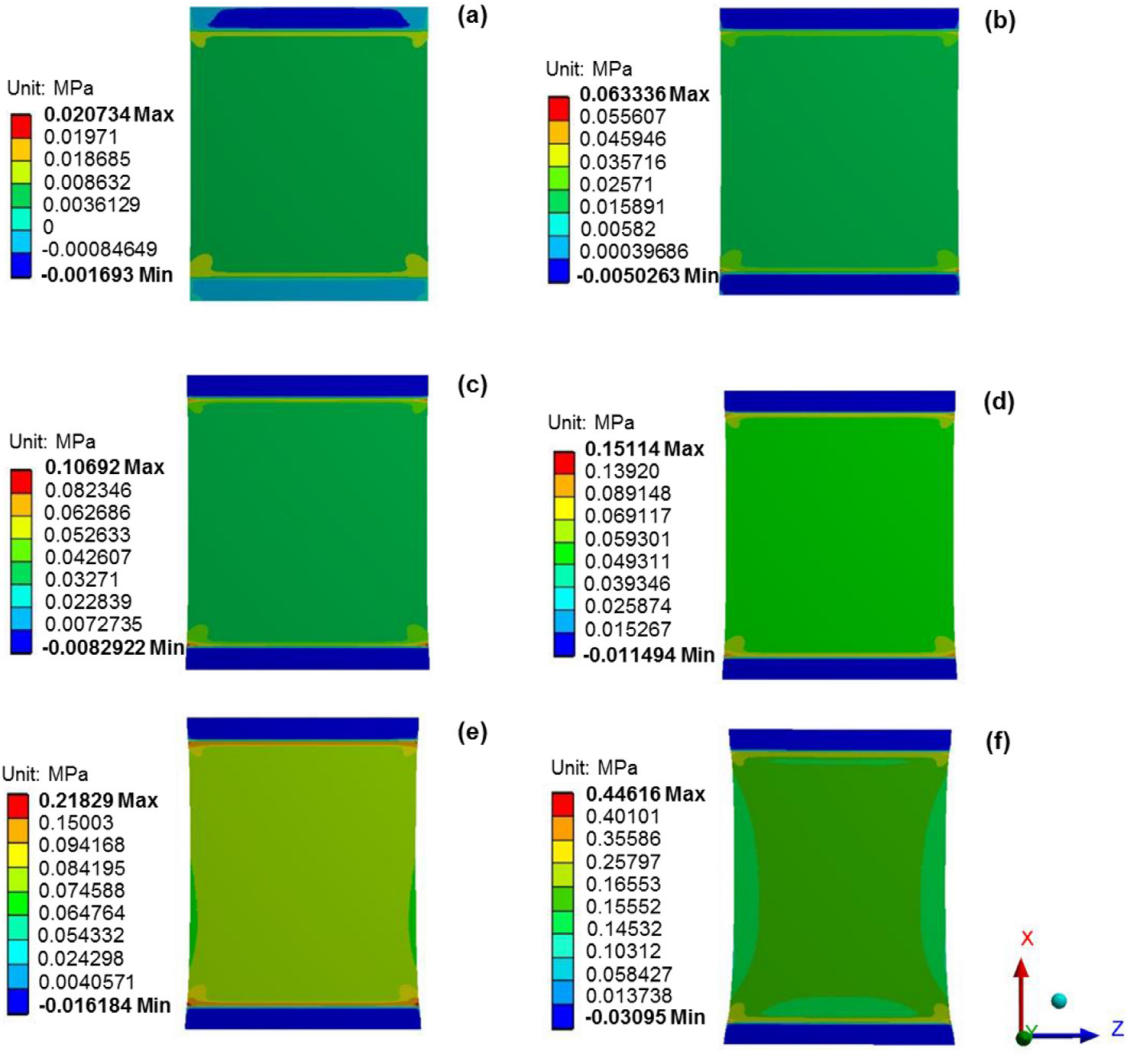
Normal tensile stress distributions on the membrane surface area under different strain loading conditions of (a) 1%, (b) 3, (c) 5%, (d) 7%, (e) 10 and (f) 20%.

**Fig. 4:**
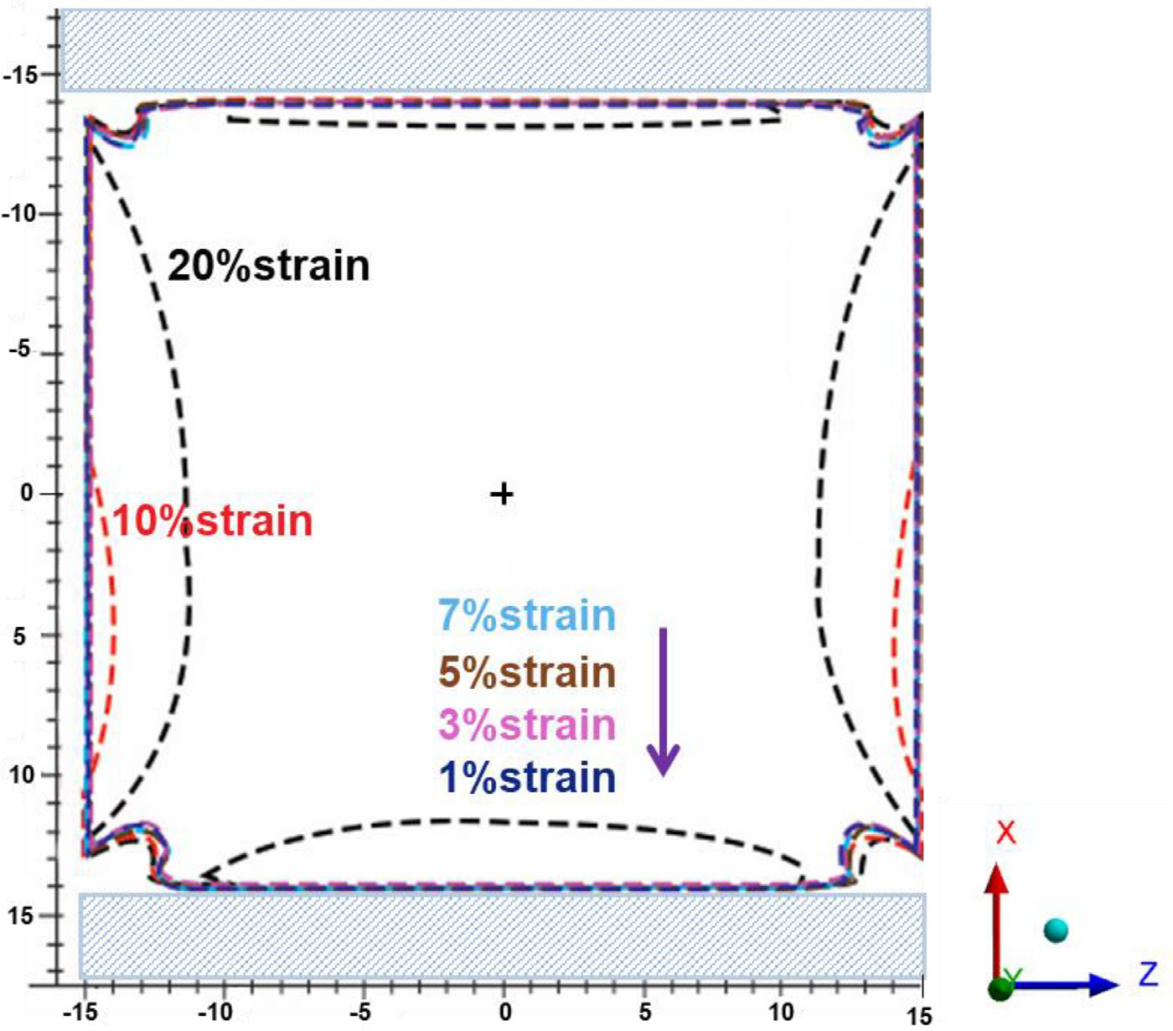
Illustration of the uniform tensile stress distributed areas.

Fig. 3(a)–(f) present the simulated normal tensile stress distribution on the membrane surface area under strain loading conditions of 1, 3, 5, 7, 10 and 20%, respectively. For the model validation, the tension force needed to maintain 20% strain loading was numerically estimated to be 3.987 N, which was 4.9% lower than that measured by the UTM (4.191 N). From Fig. 3, the areas having uniform tensile stress distribution, which was suggested for cell and tissue culture, were overlaid and illustrated in Fig. 4. The range of normal stress and the dimension of the suggested areas for cell and tissue culture under different strain loading conditions are presented in Table 2.

**Table 2:** Range of normal tensile stress and dimensions of the suggested areas under different strain loading conditions.

Displacement in *X* axis	Normal tensile stress (MPa)	Suggested area (X Z from the center of the membrane, mm)
0.29 mm (1%strain)	0.003613–0.008632	±12 · ±12
0.87 mm (3%strain)	0.01589–0.02571	±12 · ±12
1.45 mm (5%strain)	0.03271–0.04261	±12 · ±12
2.03 mm (7%strain)	0.04931– 0.05930	±12 · ±12
2.90 mm (10%strain)	0.07459–0.08419	±12 · ±12
5.80 mm (20%strain)	0.15552–0.16553	±12.25 · ±11

## 2 Experimental design, materials, and methods

The membrane used for cell culture and tensile stress loading on the cells was cast from Polydimethysiloxane (PDMS) (Silastic® T-4, Dow Corning) at the Research Unit of Inorganic Tissues, Faculty of Dentistry, Chulalongkorn University. After casting, the membrane was treated with plasma and UV radiation, and coated with gelatin, respectively. The plasma treatment condition was 60 kV, 300 Hz for 1 min using a dielectric barrier discharge (DBD) plasma generator. The UV lamp was exposed in a laminar air flow cabinet, BH2000 (Clyde-Apac, Adelaide, Australia) and the UV treatment condition was 400 mW/m^2^ for 30 min. The membrane was then coated with a 0.1% gelatin solution of the Di-Coat Corporation and left to dry on a shaker for 10 h at the room temperature.

For the tensile tests using a UTM (Instron 3360 series), the membrane was cast in a dumbbell shape with dimensions based on ASTM D638 Type IV and ASTM D412-92 standards [3,4]. A uniaxial cyclic tensile test was conducted under the ASTM D638 standard in order to obtain the stress-strain relationship of the membrane. The tests were conducted at a room temperature of 25 °C. The extension rate of the tensile test was 100 mm/min; the specimens were stretched to 25 mm. Before collecting data from the tensile test, the specimens were treated under the same cyclic loading conditions for 5 cycles in order to remove the Mullins effect [5]. In total, 30 different specimens were tested.

In an FEA of an isotropic and incompressible hyperelastic material, the nonlinear elastic deformation is explained by the constitutive laws, which is expressed as a function of the strain energy density (W) as follows [6]:

(1) $$W=W\left(\lambda_{1},\lambda_{2,}\lambda_{3}\right)$$

where *λ_i_* is the principal stretch ratio and *i* is the number 1, 2, and 3 referred to the x, y, and z dimensions, respectively. The 2nd order polynomial model was selected for describing the mechanical behavior of the membrane due to its coincidence with the tensile test data (see Fig. 1). The 2nd order polynomial model can be expressed as

(2) $$W=\sum_{i+j+k=1}^{N}C_{ijk}{\left(I_{1}-3\right)}^{i}{\left(I_{2}-3\right)}^{j}{\left(I_{3}-3\right)}^{k}$$

where *C_ijk_* is the material constant which is obtained from fitting, *i, j, k* are 0,1,2 and the strain invariants (*I*) are defined as

(3) $$\begin{array}{c} I_{1}=\lambda_{1}^{2}+\lambda_{2}^{2}\lambda_{3}^{2}\\ I_{2}=\lambda_{1}^{2}\lambda_{2}^{2}+\lambda_{2}^{2}\lambda_{3}^{2}+\lambda_{3}^{2}\lambda_{1}^{2}\\ I_{3}=J=\lambda_{1}\lambda_{2}\lambda_{3} \end{array}$$

For an incompressible material, *J* = 1, and the 2nd order polynomial model is then derived as

(4) $$W=C_{10}\left(I_{1}-3\right)+C_{01}\left(I_{2}-3\right)+C_{11}\left(I_{1}-3\right)\left(I_{2}-3\right)+C_{20}{\left(I_{1}-3\right)}^{2}+C_{02}{\left(I_{2}-3\right)}^{2}$$

To identify those material constants correctly, the data of stress-strain curves from the equi-biaxial testing and the planar testing should also be included in the fitting. Both data were calculated from the data obtained from the uniaxial tensile test using the Turner and Brennan's model [2].

The stress (*σ*) of a hyperelastic material can be derived from the strain energy density as

(5) $$\sigma =\frac{\partial W}{\partial \lambda }$$

In the FEA simulation, the geometry of the membrane model was rectangular and had a dimension of 30 mm x 35 mm x 1 mm (Fig. 2a). This was the real shape and dimension when it was used with the linear tension loading apparatus for cell culture (Thai patent ID: 1401007155) [1]. The model had 108,000 computational hexahedral elements. The material constants obtained from the fitting with the experimental data (Table 1) were used as input for the mechanical properties of the membrane in ANSYS WORKBENCH R16.2. Boundary conditions were set based on the practical operating conditions of the strain loading on cells. Each side of the membrane with a width of 3 mm was attached to stainless steel plates. One side was held stationary and thus the displacement B was fixed in *x*-axis, and the displacement C was fixed in all axes (Fig. 2b). On the other side, the membrane was stretched in *x*-axis to apply tensile stress on cells, and thus the displacement A was set to 0.29, 0.87, 1.45, 2.03, 2.90, and 5.80 mm to simulate the strain loading conditions of 1, 3, 5, 7, 10, and 20%, respectively.

Model validation was done by comparing the tension force required to generate a 20% strain loading experimentally and numerically. The simulation data were presented in terms of tensile stress distribution. The membrane area having uniform tensile stress distribution was defined as the area having local tensile stresses varied within the range of 0.01 MPa approximately, and was suggested for cell culture.

## Declaration of Competing Interest

The authors declare that they have no known competing financial interests or personal relationships which have, or could be perceived to have, influenced the work reported in this article.

## References

[1] Tantilertanant Y., Niyompanich J., Everts V., Supaphol P., Pavasant P., Sanchavanakit N. (2019). Cyclic tensile force stimulates BMP9 synthesis and in vitro mineralization by human periodontal ligament cells. J. Cell Physiol..

[2] Turner D.M., Brennan M. (1990). The multiaxial elastic behaviour of rubber. Plast. Rubber Process. Appl..

[3] American Society for Testing and Materials standards, Test method for tensile properties of plastics. Designation: D638-02a.

[4] American Society for Testing and Materials standards, Vulcanized rubber and thermoplastic rubbers and thermoplastic elastomers - tension: D412-92.

[5] Cantournet S., Desmorat R., Bessona J. (2009). Mullins effect and cyclic stress softening of filled elastomers by internal sliding and friction thermodynamics model. Int. J. Solids Struct..

[6] ANSYS® Fluent, Release 16.2, ANSYS Workbench Help, ANSYS, Inc., USA.

